# CSI shark edition: revealing illegal trade with DNA

**DOI:** 10.1093/conphys/coy022

**Published:** 2018-05-03

**Authors:** Taryn D Laubenstein

**Affiliations:** ARC Centre of Excellence for Coral Reef Studies, James Cook University, Townsville, Queensland 4810, Australia

Imagine you are a fisheries enforcement officer in Brazil. Your job is to monitor the capture and trade of fish, ensuring that fishers adhere to laws on overfishing and threatened species. But you have a thorn in your side: illegal trading of sharks.

Once passed over for other, more familiar fishes, sharks have become a major target thanks to the demand for shark fins in Asian countries to make shark fin soup. A shark fin weighing 1 kilogram could sell for $1000 USD in China, making it a lucrative target for fishers. Harvesting of sharks has skyrocketed, and many shark species, hampered by low fertility and late sexual maturity, have experienced drastic population declines. Governments and international organizations have recognized the severity of these declines, and consequently categorized a number of species as ‘endangered’ and banned their harvest.

This is where you step in: to enforce bans on shark harvest and trading.

However, you have a problem: fishers know about the bans, but they still want to turn a profit. So sometimes, by the time sharks arrive at local fish markets, the features that would help you identify the species, like the fins or heads, have been removed. How can you do your job and identify banned sharks?

Leonardo Feitosa *et al*. (2018) have proposed a solution known as DNA barcoding. Just as a scanner at the grocery store can discern between milk and eggs with the barcode on the packaging, so too can scientists distinguish between shark species using short sequences of DNA. This technique is common for species identification in a wide range of organisms. However, Feitosa and colleagues refined the method by using two DNA sequences, instead of the usual one, to identify a broader range of shark species.

To test their method, Feitosa’s team scoured local fish markets, landing ports, and fishery inspection agencies across Brazil’s North Coast, looking for shark specimens that were not visually identifiable. They also collected samples that were harvested accidentally as bycatch from industrial shrimp trawlers. In total, they acquired 427 shark samples over a 2-year period.

Using their DNA barcodes, the team identified 17 different shark species being harvested and traded. More than half of those species are already categorized as threatened under international and Brazilian laws. These results are troubling; if harvest of these endangered species continues, their populations could dwindle to dangerously low numbers. Particularly at risk are the endemic species, those with very restricted geographic ranges, which could face extinction if harvest continues.

While their results are sobering, Feitosa and colleagues have also provided hope for fisheries enforcement officers like you. Armed with DNA barcoding methods, you are now one step closer to effective management of shark fisheries in Brazil. It’s not an easy task—Brazil’s North Coast is considered both a hotspot for shark conservation and a key fishing zone for local fishers, so conflicts are bound to arise. But when there is evidence of illegal trade of endangered shark species, you have the CSI skills to crack the case.

**Figure:**
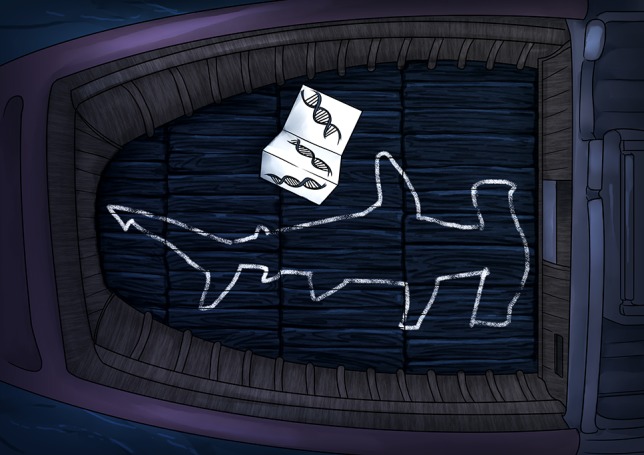


Illustration by Erin Walsh; Email: ewalsh.sci@gmail.com
